# Plasma Vascular Biomarkers and Cognitive Resilience in APOE ε4 Carriers: A Longitudinal Analysis

**DOI:** 10.1002/alz70861_108924

**Published:** 2025-12-23

**Authors:** Mohamed Hosni Kamel Elkellawi, Mohamed Khaled Ali Mohamed Ali Zid, Arwa Ayman Nagah Ahmed Shahin, Asmaa Gamal Abdelmoez Hassanein Bassiouni, Ahmed Hussein Mohamed Ismail Kira

**Affiliations:** ^1^ Cairo University Faculty of Medicine, Cairo, Cairo Egypt; ^2^ Cairo University Faculty of Medicine, Cairo, 11553 Egypt

## Abstract

**Background:**

The Apolipoprotein E Epsilon 4 (APOE ε4) gene represents the most significant genetic risk factor for developing late‐onset Alzheimer’s disease, yet some carriers maintain their cognitive function as they age. The mechanisms behind this resilience have not been identified. Research indicates that vascular health acts as a protective factor but its connection to ε4 carrier resilience particularly through plasma biomarkers has not been fully explored.

**Method:**

We analyzed longitudinal data from the Alzheimer’s Disease Neuroimaging Initiative (ADNI), which included cognitively normal older adults (≥65 years) who had at least 3 years of cognitive follow‐up and APOE genotyping. The researchers measured vascular markers (VCAM‐1, ICAM‐1, homocysteine, angiopoietin‐2, sFlt‐1) in plasma samples at the beginning of the study. The participants received APOE ε4 status and cognitive trajectory classification into resilient (no decline) and non‐resilient (decline) groups. The analysis used mixed‐effects models to examine biomarker relationships with cognitive outcomes while adjusting for age, sex, education and vascular risk factors.

**Result:**

Among 300 APOE ε4 carriers, 120 showed stable cognitive function over 3+ years. The resilient carriers showed lower initial VCAM‐1 and angiopoietin‐2 levels (*p* <0.01), matching earlier research about vascular endothelial dysfunction causing cognitive decline. The research showed that VCAM‐1 levels had a stronger positive relationship with preserved cognition in ε4 carriers than in non‐carriers which supports the idea that vascular inflammation worsens APOE ε4‐related neurodegeneration. The results maintained their statistical significance after controlling for comorbidities which supports the use of vascular biomarkers to detect people with cognitive resilience who have genetic risk factors.

**Conclusion:**

Specific plasma vascular biomarkers may identify APOE ε4 carriers with cognitive resilience, suggesting vascular pathways play a role in offsetting genetic risk. These findings support incorporating vascular health profiling in personalized dementia prevention strategies.

